# Neurology Case Report: Rapidly Progressive Dementia and Extrapyramidal Symptoms as the First Presentation of Leptomeningeal Carcinomatosis

**DOI:** 10.7759/cureus.22923

**Published:** 2022-03-07

**Authors:** Madihah Alhubayshi, Dinah Alasmari, Bashaer Almahdi, Osama Khojah, Faris Allaf, Hatim Q AlMaghrabi

**Affiliations:** 1 Neurology, National Guard Hospital, King Abdulaziz Medical City, Jeddah, SAU; 2 College of Medicine, King Saud bin Abdulaziz University for Health Sciences, Jeddah, SAU; 3 Anatomical Pathology, National Guard Hospital, King Abdulaziz Medical City, Jeddah, SAU

**Keywords:** rapid progressive dementia, extrapyramidal symptoms, lung cancer, brain tumor, leptomeningeal metastasis, carcinomatous meningitis, leptomeningeal carcinomatosis

## Abstract

Leptomeningeal carcinomatosis (LC) is a rare complication of primary malignancy that spreads to leptomeninges and cerebrospinal fluid (CSF). Due to its rarity, it is often diagnosed as a late complication of an advanced tumor. This report presents a case study of a 72-year-old nonsmoking female with multiple comorbidities with two-week rapidly progressive cognitive decline and extrapyramidal symptoms (EPS). She presented with speech difficulties, tension headaches, and episodes of inattention. On examination, she had a masked face, mild bradykinesia, mild rigidity more apparent in the limbs than axially, and slight hyperreflexia in the lower limbs with a normal plantar reflex (down-going). Magnetic resonance imaging (MRI) of the brain with gadolinium showed diffuse leptomeningeal dissemination. CT of the right lower lobe showed lobe apical segment mass lesion with air bronchogram extension to the hilum, which raised the suspicion that the patient had lung cancer. The microscopic analysis of cerebrospinal fluid (CSF) cytology showed poorly differentiated malignant cells favoring adenocarcinoma. Based on these investigations, leptomeningeal dissemination on the MRI led to a wide differential diagnosis; however, given the findings in the CT scan and CSF, the patient was diagnosed with leptomeningeal carcinomatosis secondary to metastatic lung cancer. Although LC is a rare terminal complication that presents with a wide range of symptoms, typically including headache, altered mental status, diplopia, back pain, cerebral signs, and leg weakness, our patient presented with an uncommon presentation, which was EPS. Therefore, this case report highlights the importance of early detection of LC in any patient presenting with unspecific neurological manifestations.

## Introduction

Leptomeningeal carcinomatosis (LC), or carcinomatous meningitis or leptomeningeal metastasis, is a rare and late-stage complication of a locally advanced or malignant tumor, which metastasis to leptomeninges and cerebrospinal fluid (CSF) [1,2]. Malignant cells, which proliferate into the leptomeninges, occur more commonly with hematological malignancies and less frequently with solid tumors, with an incidence of 5%-15% and 5%-8%, respectively [1]. Among the reported solid tumors that were associated with LC, cancer of unknown primary, breast cancer, lung cancer, and melanoma were the most reported [3]. Nevertheless, LC has been documented to be related to many systemic cancer types, including cancer of the ovary, fallopian tube, cervix, prostate, urinary bladder, gallbladder, stomach, and kidney and cutaneous melanoma [4]. The clinical presentation of LC depends on the location of involvement, so the disease causes a wide range of nonspecific manifestations [2]. Signs and symptoms are usually related to meningeal irritation, cranial and spinal nerve dysfunction, and increased intracranial pressure [1]. Patients complain of altered mental status, headache, back pain, cerebral signs, and weak legs [5]. Some patients may also develop multiple cranial neuropathies or rapidly progressive dementia [1]. The diagnosis of LC is challenging and requires a high index of suspicious by the clinicians [2]. The diagnosis often requires a combination of magnetic resonance imaging (MRI) of the brain and spine and cerebrospinal fluid analysis with cytology [1]. Since extrapyramidal symptoms (EPS) are not a common presentation of LC, here, we describe a case of an elderly female with rapidly progressive cognitive decline and EPS due to LC secondary to lung adenocarcinoma.

## Case presentation

A 72-year-old nonsmoking female was diagnosed with hypertension, osteoporosis, and hypothyroidism. She presented to the emergency department in December 2020 and had rapidly progressive dementia for one month. According to the family, during the previous month, the patient experienced memory loss, progressive disorientation, speech difficulty, tension headache, and episodes of inattention associated with a delayed response, but the patient was responding to verbal and painful stimuli. Before that, she was independent and without cognitive impairment. She used to pray the five prayers on time, but during the previous month, she became unaware of the prayer time. The patient experienced a weight loss of 20 kg in three months. She did not have a fever, vomiting, loss of consciousness, or fatigue. She did not have a prior history of seizures. On clinical evaluation, she was disoriented to time, but not to place or person. On her mini-mental state examination, she scored 17/30, but she was illiterate. On neurological examination, she had a masked face, mild bradykinesia, mild rigidity more apparent in the limbs than axially, and hyperreflexia (+3) in the lower limbs bilaterally with absent Babinski sign and no ankle clonus bilaterally. She had symmetrical strength bilaterally with no pronator drift. She did not have nuchal rigidity, ataxia, or cranial nerve deficit. The patient was admitted for further investigations on suspicions of recurrent focal seizure with impaired awareness or nonconvulsive seizure.

Cerebrospinal fluid (CSF) analysis showed normal cell count and total protein, while her glucose levels were slightly elevated (5.2 mmol/L). Hence, the antibiotics were discontinued. Electroencephalography (EEG) suggested a mild cognitive impairment since it showed a diffuse slowing of the background frequencies, typically slowing the posterior dominant rhythm and reducing the beta activity in the frontal regions. Theta waveforms increase in abundance along with delta activity. The previous findings were intermittent and then progress to being more continuous, and as they became more continuous and slower, the patient became more confused.

**Figure 1:**
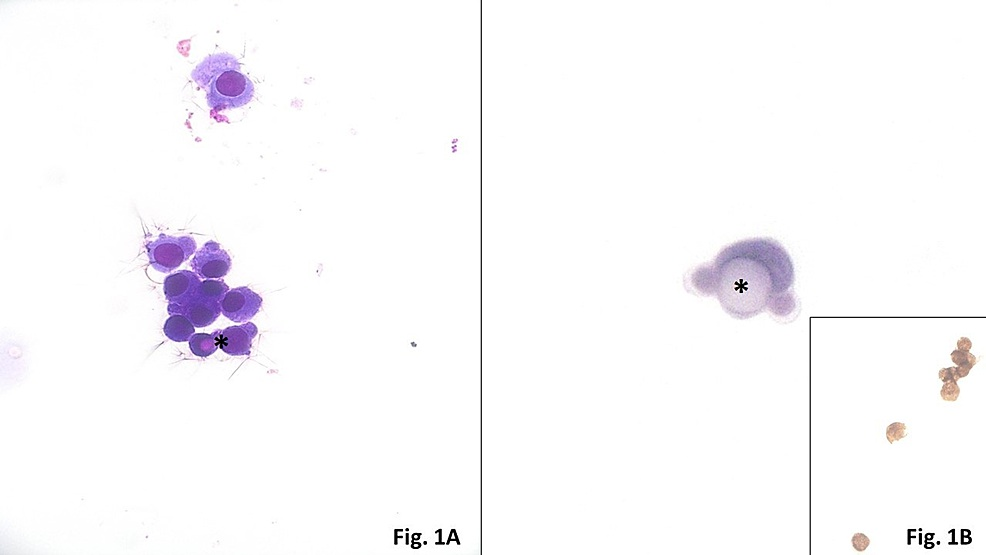
Microscopic analysis of CSF cytology 1A–1B: Groups of relatively cohesive adenocarcinoma cells with irregular nuclear membrane, high N:C ratio, and vacuolated cytoplasm that contains mucin. Asterisks highlight mucin vacuoles. Inset shows immunocytochemistry performed on CSF cytospin material demonstrating brown staining (positive) nuclear immunoreactivity of carcinoma cells for antibodies against thyroid transcription factor 1 (TTF1) (antibody clone SP141, BenchMark XT, Ventana, Tucson, USA). 1A: Diff-Quick stain, air-dried cytospin, ×600. 1B: Pap stain, ethanol-fixed cytospin, ×600.

Microscopic analysis of CSF cytology showed the presence of poorly differentiated malignant cells in favor of adenocarcinoma (Figure 1A, 1B).

**Figure 2:**
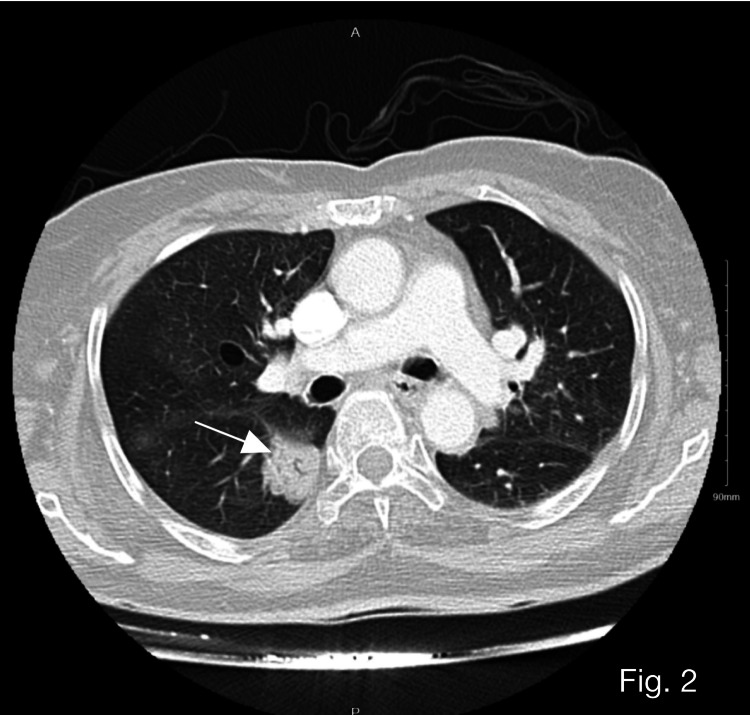
CT of the chest, abdomen, and pelvis (CAP-CT) showing a right lower lobe apical segment mass lesion with air bronchogram extension to the hilum

Differentials for the primary origin of the cells included the lungs, gastrointestinal tract, or gynecological malignancies. CT of the chest, abdomen, and pelvis (CAP-CT) revealed right lower lobe apical segment mass lesion with air bronchogram extension to the hilum with differential diagnosis including invasive adenocarcinoma or lymphoma (Figure 2).

**Figure 3:**
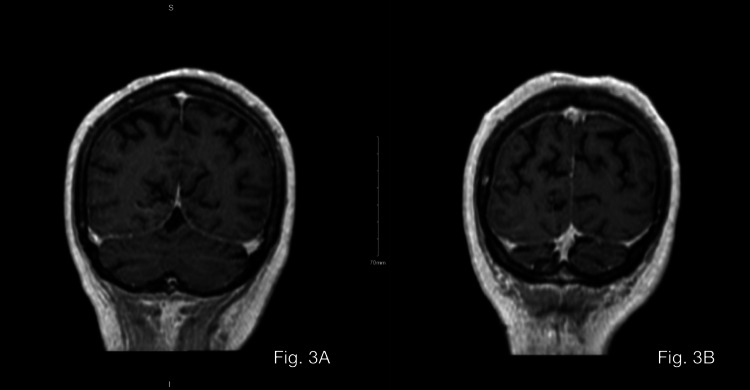
Brain T1-weighted MRI 3A–3B: Brain T1-weighted MRI (coronal view) demonstrating some cortical foci of bright T1 signal intensity with minimal blooming effects of gradient images. Also, there is diffuse cortical and leptomeningeal enhancement.

Magnetic resonance imaging (MRI) of the brain with gadolinium showed diffuse leptomeningeal dissemination that is characteristic of adenocarcinoma (Figure 3A, 3B).

Subsequently, she was diagnosed with leptomeningeal carcinomatosis due to suspicion of lung cancer metastasis.

Based on the radiological and CSF evidence, the patient’s case was evaluated as stage IV lung cancer. The patient was given an Eastern Cooperative Oncology Group (ECOG) performance status of 3, which excluded curative chemotherapy and immunotherapy eligibility. Palliative radiotherapy evaluation showed that it would have no benefit. Therefore, she was placed on symptomatic palliative care and received a course of dexamethasone. Thirteen days post-admission, the patient was discharged to her home and followed up by the oncology and palliative teams. The neurology team examined the patient again 32 days after discharge. Due to the insufficient amount and low quality of the first tests, repeated LP and CSF analyses were done to confirm the presence of malignant cells, and immunostaining for thyroid transcription factor 1 (TTF1) was positive. She was fluent in speaking and comprehensive and obeyed commands yet disoriented to time and place. Eventually, the patient was discharged since she was stable neurologically.

## Discussion

Approximately 5%-18% of lung cancer cases are associated with LC [6]. The period ranges from two days to eight years from diagnosis of a lung malignancy to diagnosis of LC [6]. The clinical presentation of LC may be as a complication of malignancy or as the first manifestation of primary cancer [7]. Diagnosing LC can be difficult as most cases are identified post-mortem [8]. There are differences in how LC patients present, which depend on the affected neuraxes. The hallmark of LC is signs and symptoms that stem from multiple levels of the nervous system. However, patients may present with one symptom or no symptoms at all, which means that a high index of suspicion should always be present [9]. Signs and symptoms are usually associated with meningeal irritation, cranial and spinal dysfunction, and/or increased intracranial pressure (Table 1) [1,10-14].

**Table 1:** Signs and symptoms of LC

Cortical signs and symptoms	Cranial nerve signs and symptoms	Cerebellar signs and symptoms	Spinal signs and symptoms
Headache	Diplopia	Gait ataxia	Nuchal rigidity
Confusion	Visual loss	Limb dysmetria	Bowel and bladder dysfunction
Lethargy	Facial sensory changes		Pain (e.g., radicular, neck, or back)
Nausea and/or vomiting	Dysphagia		Limb weakness
Seizures	Dysarthria		Sensory changes
Weakness	Changes in taste		Reflex abnormality
Sensory changes	Hearing loss		Cauda equina syndrome
Apraxia	Facial weakness		
Delirium	Tinnitus		
Dementia	Vertigo		
Hydrocephalus (communicating or obstructive)	Dysphonia		
Extrapyramidal symptoms (e.g., tremors)			

However, Naydenov and Taylor hypothesize that symptoms are not heterogeneous but rather a result of one or a combination of an increase in the intracranial pressure, parenchymal irritation associated with inflammatory cytokines, and clumping at the rootlets [15]. They found that 99% of patients fall into one of these categories, and our patient falls under many of these categories [15]. As a result of this disease, patients complain of general symptoms such as altered mental status, back pain, nausea and vomiting, cerebellar signs, diplopia, leg weakness, and most commonly headache [7]. Our patient suffered from rapid progressive dementia (RPD) with extrapyramidal symptoms (EPS).

EPS includes disturbances in movement, tone, and/or posture, dystonia, parkinsonian signs, tardive dyskinesia, and tardive akathisia [16,17]. EPS occurs due to disturbances in the dopaminergic neurons in the central nervous system [18]. It also commonly occurs with medication use, especially with the use of antipsychotic medications such as haloperidol, risperidone, olanzapine, chlorpromazine, and prochlorperazine. EPS has been reported in up to 60% of patients taking long-term antipsychotics [19]. Ishiki et al. investigated the prevalence of EPS in cancer patients undergoing palliative therapy [18]. They found that 14.8% of patients with EPS had metastasis to the brain, although some of them may have taken medications that caused the EPS [18]. The occurrence of EPS with LC has previously been reported; in the case reported by Francolini et al., his patient suffered from rapid cognitive decline, gait disturbances, postural instability, and dysarthria [20]. RPD is an uncommon presentation of LC. Cases of RPD in association with LC were previously reported by Muangpaisan, who reported two cases, and Francolini et al. and Jadav et al., both of whom reported one case [7,20,21]. Muangpaisan reported two cases of RPD in patients who were later diagnosed with LC due to gastric cancer [7].

Similar to our patient, LC patients tend to present acutely with neurological changes ranging from days to weeks [7]. The prognosis for RPD varies based on the underlying cause of the presentation. Diagnosing reversible RPD is crucial because if there is any delay in treatment, it can cause irreversible functional impairment or even death. One reversible differential diagnosis of RPD that might be underestimated is autoimmune dementia.

Autoimmune dementia has been defined as one or a combination of the following categories: syndromic class (e.g., progressive encephalomyelopathy associated with myoclonus and rigidity), eponymous class (e.g., Morvan’s fibrillary chorea), serology class (e.g., encephalopathy with voltage-gated potassium channel antibody), or pathological class [22,23]. Thus, to exclude the reversible causes, it is important to perform investigations that include hematological and chemical laboratory studies and neural antibody markers. Also, oncological evaluation of serum and CSF testing should be conducted in the case of positive neural antibody markers that raise the suspicion of paraneoplastic causes [24]. In addition, to diagnose some cases, brain imaging, EEG, and brain biopsy are necessary [7]. All previous investigations will assist in the early detection and management of reversible causes such as autoimmune dementia.

The main modalities for the diagnosis of LC are MRI, CSF analysis, or both, with variable sensitivity and specificity depending on the primary source of metastasis (70% sensitivity and 77%-100% specificity) [1]. MRI with abnormal features (leptomeningeal enhancement, subependymal deposits, and hydrocephalus) with suspicion signs and symptoms is often enough to diagnose LC. MRI alone can be enough to diagnose LC in solid tumors because the attached solid metastases to the natural network with a nodular pattern can be visible by MRI [1,9]. However, MRI’s sensitivity often falls short, and CSF is required to adjunctly confirm the diagnosis. With the importance of CSF rising in hematopoietic primary tumors, it may reveal pleocytosis, increased protein content, and hypoglycorrhachia. It is described as a gold standard confirmatory test, with serial sample testing having a specificity of more than 95% [1,3]. More often, MRI and CSF are used concurrently to complement the diagnosis of LC (66%-76% higher compared with a single LP) [3,4,9].

The diagnosis of primary cancer can be sometimes challenging as in our case. To optimize sensitivity and avoid performing unnecessary or invasive procedures, the ideal sequence of interventions and studies in a particular patient requires a careful judgment of the probable reliability of a variety of presumptive diagnostic concerns. To reach the diagnosis of lung cancer, clinicians must follow the guidelines that were established based on multiple published data where the focus was on the quality and the net benefit [25]. If there was a high suspicion of metastasis that was detected on imaging, clinicians can decide the tissue site selection for the confirmation of metastasis based on the ease of entry and the risk of operation. According to the guidelines, in patients who are suspected to have lung cancer based on imaging and have solitary extrathoracic or multiple distant metastases, the diagnosis can be reached using the easiest and safest procedure, such as fine-needle aspiration and tissue biopsy [25]. In our case, based on the suggestive lung CT and brain MRI findings, CSF analysis was evaluated to be the most appropriate test, showing a group of relatively cohesive adenocarcinoma cells and thus establishing the diagnosis of primary lung cancer with LC.

The goal of treating LC is to prolong survival, but the outcomes of LC are not favorable as most cases, which do not receive treatment pass away within about a month, and most cases pass away within less than a year even after receiving radiation and/or chemotherapy [7]. LC is treated with intrathecal chemotherapy. The two cases reported by Muangpaisan and the case reported by Francolini et al. all died within a month of their admission [7,20]. However, our patient did not receive any curative treatment during her hospital stay and was not deemed fit enough for palliative radiotherapy, yet she did not further deteriorate.

## Conclusions

Leptomeningeal carcinomatosis is an uncommon fatal complication of lung cancer, yet it is an established oncological condition. Presenting with a wide variety and acuity of neurological symptoms, including RPD and EPS, LC carries a poor prognosis. Diagnosis of LC can be elusive and is often reached post-mortem; as such, a high degree of suspicion is warranted for oncology patients presenting with RPD and EPS among other neurological signs and symptoms. LC manifestations and diagnosis remain an understudied subject that warrants further research to expedite the management of patients affected by it and improve their quality of life.
